# A high‐throughput bone marrow 3D co‐culture system to study resistance to BCR signalling targeted agents in B‐NHL

**DOI:** 10.1111/bjh.70273

**Published:** 2025-12-08

**Authors:** Alex Zadro, Alberto Arribas, Maria Vittoria Colombo, Eleonora Cannas, Filippo Spriano, Luciano Cascione, Afua Adjeiwaa Mensah, Federico Simonetta, Dalila Petta, Christian Candrian, Chiara Arrigoni, Francesco Bertoni, Matteo Moretti

**Affiliations:** ^1^ Regenerative Medicine Division, Institute for Translational Research Faculty of Biomedical Sciences, Università Della Svizzera Italiana (USI)—Ente Ospedaliero Cantonale (EOC) Bellinzona Switzerland; ^2^ Institute of Oncology Research, Faculty of Biomedical Sciences USI Bellinzona Switzerland; ^3^ Swiss Institute of Bioinformatics Lausanne Switzerland; ^4^ Division of Hematology, Department of Oncology Geneva University Hospitals, University of Geneva Geneva Switzerland; ^5^ Translational Research Centre in Onco‐Hematology, Faculty of Medicine University of Geneva Geneva Switzerland; ^6^ Service of Orthopedics and Traumatology, Department of Surgery Ente Ospedaliero Cantonale (EOC) Lugano Switzerland; ^7^ Euler Institute Faculty of Biomedical Sciences, USI Lugano Switzerland; ^8^ Oncology Institute of Southern Switzerland Ente Ospedaliero Cantonale (EOC) Bellinzona Switzerland; ^9^ IRCCS Istituto Ortopedico Galeazzi Cell and Tissue Engineering Laboratory Milan Italy

**Keywords:** BCR signalling targeted agents, in vitro 3D bone marrow model, non‐Hodgkin B cell lymphomas

## Abstract

Bone marrow (BM) involvement in B‐cell non‐Hodgkin lymphoma (B‐NHL) is associated with poor prognosis, as the BM microenvironment provides a protective niche that promotes therapeutic resistance. We developed a simplified, automated and high‐throughput 3D BM co‐culture model that faithfully reproduces key tumour–stroma interactions. In our system, BM stromal cells (BMSCs) decreased lymphoma cell sensitivity to Phosphatidylinositol 3‐kinase (PI3K) and BTK inhibitors. Moreover, we show that our 3D platform enables the mechanistic studies of microenvironment‐mediated drug resistance and has the potential to be developed into a tool for personalized therapeutic strategies for B‐NHL.

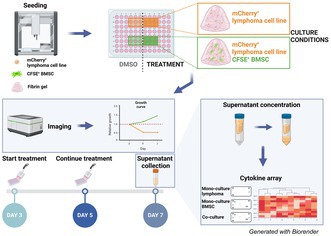

The central role of B‐cell receptor (BCR) signalling in the pathogenesis of B‐cell non‐Hodgkin lymphomas (B‐NHL) makes it a key target for therapeutic intervention.^1^ Notwithstanding the number of therapeutic options available, resistance to treatments remains a significant clinical challenge in managing B‐NHL patients.^2^, ^3^, ^4^ In this context, the bone marrow (BM) microenvironment has been described as a ‘privileged’ niche where cancer cells can evade therapeutic interventions, particularly chemotherapy.^5^, ^6^, ^7^ Considering that available preclinical models are not suited to investigate the influence of the BM tumour microenvironment (TME) on B‐NHL therapeutic resistance to BCR signalling inhibitors, we developed a high‐throughput in vitro 3D BM model to study these resistance mechanisms.

Several lymphoma cell lines were cultured in 3D fibrin gel to mimic the soft BM extracellular matrix, alone or with primary bone marrow stromal cells (BMSCs), after automated seeding performed with a liquid handler. The automated seeding procedure was highly reproducible, as evidenced by the uniform cell spreading across wells and similar distributions of measured area values between mono‐ and co‐culture conditions (Figure S1A,B). The coefficient of variation between different wells was less than 10% and not affected by culture conditions (Figure S1C).

In the presence of BMSCs, the lymphoma cell lines VL51 and SSK41 formed bigger clusters, suggesting that BMSCs influence how lymphoma cells grow in 3D fibrin gel (Figure 1A, Figure S1D). Upon exposure to different concentrations of the Phosphatidylinositol 3‐kinase (PI3K) inhibitor copanlisib, a dose–response effect was observed in the monoculture but not in the co‐culture, both in VL51 and SSK41 cells (Figure 1A–C, day 3 images in Figure S2A). The concentration of 100 nM copanlisib was selected for further studies based on its efficacy in monoculture and minimal toxicity to BMSCs, as observed at 500 and 1000 nM concentrations (Figure SB). The presence of BMSCs significantly reduced the sensitivity of VL51 and SSK41 to 100 nM copanlisib at day 7 (Figure 1D,E). In addition, the presence of BMSCs significantly reduced VL51 and REC‐1 sensitivity to the BTK inhibitor ibrutinib, and a similar trend was observed in SSK41, Karpas1718 and TMD8 models (Figure S3A, ibrutinib concentrations indicated in Table S1). The TMD8 cell line exhibited the smallest resistance to ibrutinib in the presence of BMSCs (Figure S3A). TMD8 is derived from a diffuse large B‐cell lymphoma (DLBCL) model, whereas the other cell lines are derived from marginal zone lymphoma (MZL; VL51, Karpas1718 and SSK41) or mantle cell lymphoma (MCL; REC‐1). Because bone marrow (BM) involvement is relatively uncommon in DLBCL compared to MZL and MCL, TMD8 cells may be less influenced by BM‐derived stromal resistance mechanisms, reflecting the distinct microenvironmental dependencies of lymphomas that primarily present in lymph nodes rather than the BM. Overall, BMSCs influenced the growth of MZL and MCL cell lines and reduced their sensitivity to BCR signalling inhibitors.

**FIGURE 1:**
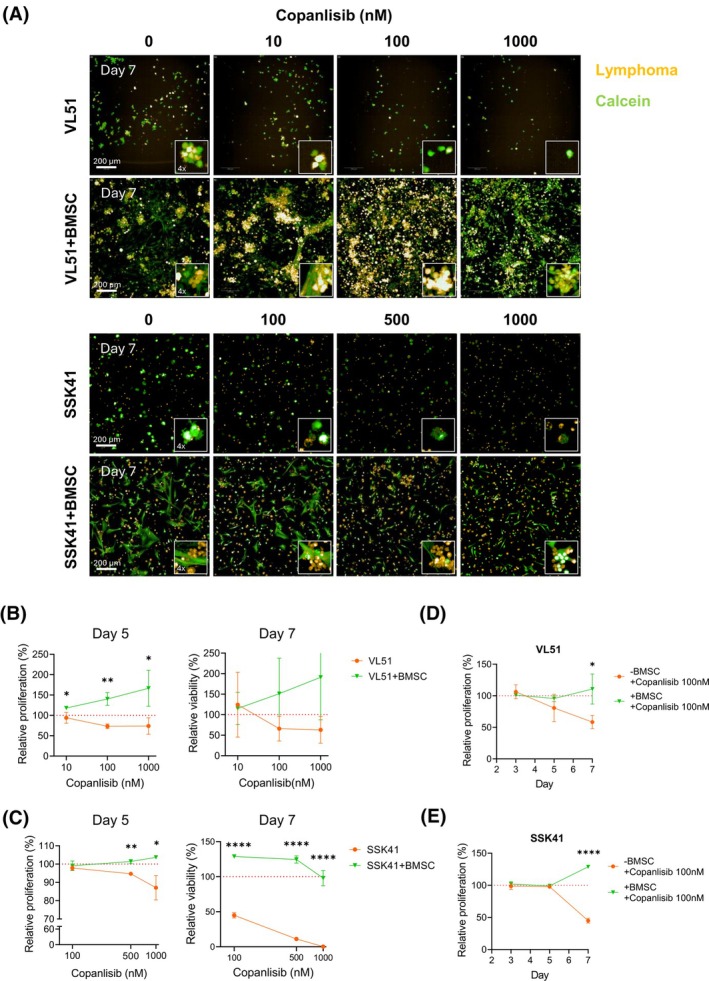
3D co‐culture with bone marrow stromal cells (BMSCs) influences the growth pattern of B‐cell lymphoma cells and reduces their sensitivity to pharmacological inhibition of PI3K and BTK. (A) Representative immunofluorescence maximum projection images of VL51 and SSK41 (in orange) cultured in the presence or absence of BMSCs upon DMSO or copanlisib (concentrations indicated in the figure) treatment on day 7. Live cells stained with calcein (in green). Images taken with 10× air objective NA 0.3, WD 5.2 mm of the Opera Phenix Plus High‐Content Screening System while keeping the plate at 37°C, 5% CO_2_ and optimal humidity. Scale bar: 200 μm, magnification: 4× objective. (B) Left, relative proliferation compared to the respective control of the VL51 cells in mono‐ or co‐culture under the different treatment conditions at day 5. Right, relative viability compared to the control of the VL51 cells in mono‐ or co‐culture under the different treatment conditions at Day 7. Values are plotted as the mean with SD of at least three independent biological replicates. Statistical significance tested with multiple *t*‐test (*p < 0.05, **p < 0.01). (C) Left, relative proliferation compared to the respective control of the SSK41 cells in mono‐ or co‐culture under the different treatment conditions at day 5. Right, relative viability compared to the control of the SSK41 cells in mono‐ or co‐culture under the different treatment conditions at Day 7. Values are plotted as the mean with standard deviation of at least three independent biological replicates. Statistical significance tested with multiple *t*‐test (*p < 0.05, **p < 0.01, ***p < 0.001, ****p < 0.0001). (D) Relative proliferation compared to the respective control of the VL51 cells in mono‐ or co‐culture upon DMSO or 100 nM copanlisib at days 3, 5 and 7. Values are plotted as the mean with standard deviation of at least three independent biological replicates. Statistical significance tested with multiple *t*‐test (*p < 0.05). (E) Relative proliferation compared to the respective control of the SSK41 cells in mono‐ or co‐culture upon DMSO or 100 nM copanlisib at days 3, 5 and 7. Values are plotted as the mean with SD of at least three independent biological replicates. Statistical significance tested with multiple *t*‐test (****p < 0.0001).

To identify potential molecules involved in the resistance mechanism, we analysed the supernatant of VL51 lymphoma cells, treated with or without 100 nM copanlisib in the presence or absence of BMSCs, for the presence of 105 secreted factors (Table S2). We identified 14 cytokines differentially secreted in the different culture and treatment conditions. Among these, we identified cytokines that were already reported in the literature to be involved in therapeutic resistance in lymphoma, such as Interleukin 6 (IL‐6)^8^ and Vascular Endothelial Growth Factor (VEGF),^9^ and in the homing of malignant cells to the bone marrow, such as CXCL12.^10^ Moreover, all the identified cytokines were also secreted by BMSCs in monoculture conditions, suggesting that BMSCs were the primary source of these cytokines (Figure 2A, whole panel in Figure S4A and membranes in Figure S4B). To evaluate whether IL‐6 contributed to drug resistance in our model, the anti‐IL‐6 monoclonal antibody sirukumab was tested in combination with copanlisib and ibrutinib. Sirukumab was first assessed as a single agent in VL51 monoculture to determine the highest non‐toxic dose. A concentration of 10 μg/mL was selected, as it showed no significant impact on VL51 viability after 4 days of treatment (Figure S5A,B). In co‐culture with BMSCs, sirukumab partially restored VL51 sensitivity to copanlisib (Figure 2B–D), and to a lesser extent to ibrutinib (Figure S5C–E), suggesting that IL‐6 contributes to, but is not solely responsible for, the resistance mechanism in this experimental setting.

**FIGURE 2:**
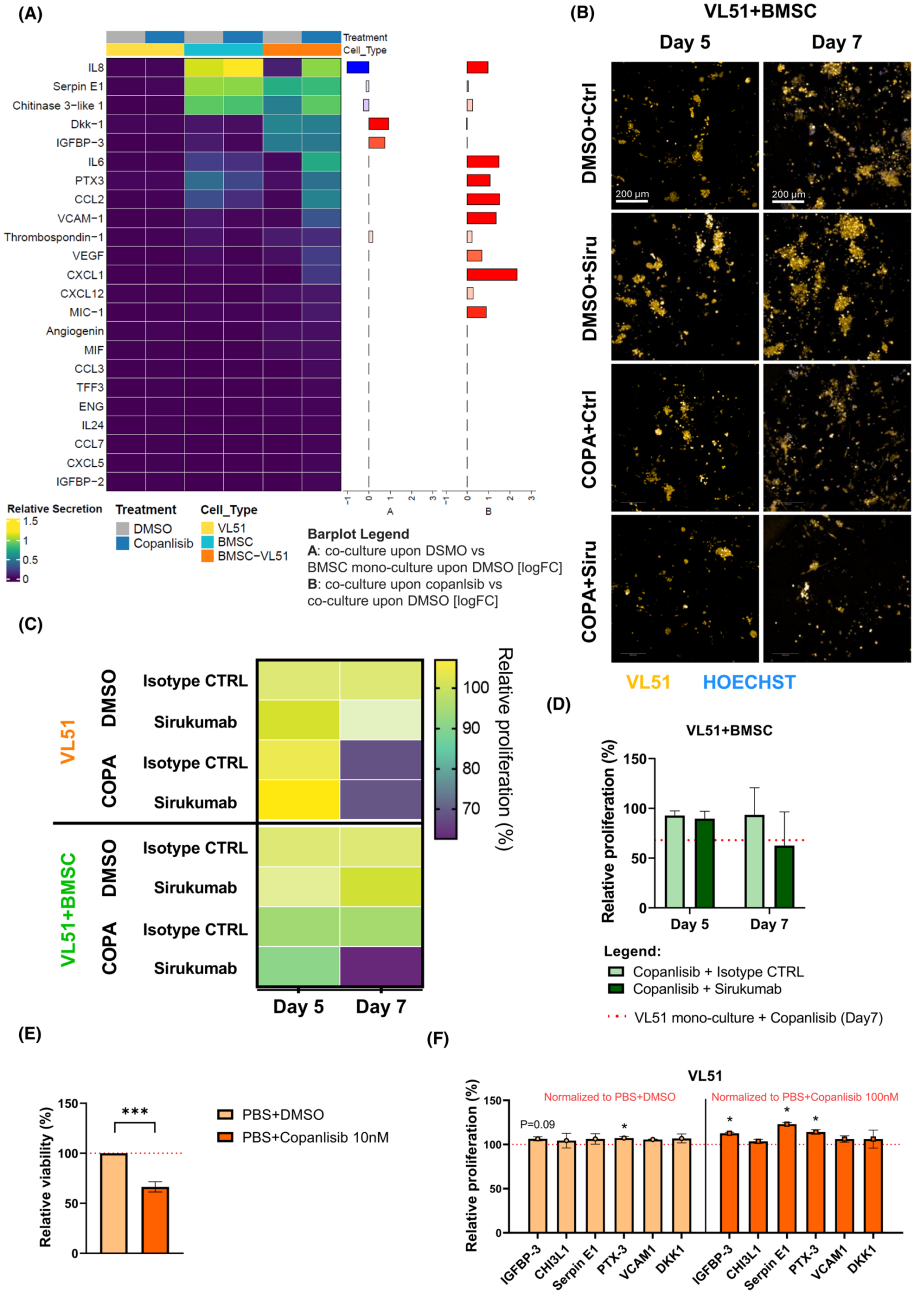
IL‐6, IGFBP‐3, Serpin E1 and PTX‐3 influence VL51 sensitivity to copanlisib. (A) Plot showing a subset of the cytokines analysed with the cytokine array. The heat map was generated with the relative secretion values (row values normalized to internal positive and negative controls) of each cytokine in the different culture conditions. The bar plots show the log fold change values comparing co‐culture upon DMSO to bone marrow stromal cells (BMSCs) monoculture upon DMSO (A) or copanlisib to DMSO conditions in the co‐culture (B). (B) Representative immunofluorescence maximum projection images of VL51 (in orange) cultured in the presence of BMSCs upon DMSO+isotype CTRL, DMSO+sirukumab, copanlisib+isotype CTRL or copanlisib+sirukumab treatment on Day 5 and 7 (copanlisib concentration = 100 nM, sirukumab concentration = 10 μg/mL). Nuclei stained with Hoechst (in blue). Images taken with 10x air objective NA 0.3, WD 5.2 mm of the Opera Phenix Plus High‐Content Screening System while keeping the plate at 37°C, 5% CO_2_ and optimal humidity. Scale bar: 200 μm. (C) Plot showing the response of VL51 in mono‐ and co‐culture to copanlisib with or without sirukumab. The heat map was generated with the relative proliferation values of each culture condition at Days 5 and 7. (D) Bar plot showing the quantification of the mCherry‐positive area on maximum projection images, representative of VL51 occupied volume. The measurements shown were normalized to the Day 3 time point, and the co‐culture‐treated conditions were normalized to the respective DMSO control. Values are plotted as mean with standard deviation of at least three independent biological replicates and represent the relative proliferation of cells in the indicated condition compared to the control. Statistical significance was tested with a multiple *t*‐test. The dotted red line represents the proliferation of VL51 in monoculture upon copanlisib+isotype CTRL at day 7 (copanlisib concentration = 100 nM). (E) Bar plot showing the effect of 10 nM copanlisib on VL51 cells viability tested with MTT assay. The plotted values represent the mean with a standard deviation of three independent biological replicates. Statistical significance tested with multiple *t*‐test (*p < 0.05, **p < 0.01, ***p < 0.001). (F) Bar plot representing the relative viability of VL51 cells tested with MTT assay under the different culture conditions. The cytokine‐stimulated conditions upon DMSO normalized to the respective Phosphate Buffered Saline (PBS) Insulin‐like growth factor‐binding protein 3 + DMSO control, representative of the cytokine‐driven proliferative advantage, are plotted in light orange. The cytokine‐stimulated conditions upon copanlisib, normalized to the respective PBS + copanlisib control, representative of the cytokines' influence on copanlisib sensitivity, are plotted in dark orange. The plotted values represent the mean with a standard deviation of three independent biological replicates. Statistical significance tested with multiple *t*‐test (*p < 0.05).

Therefore, to identify new potential secreted resistance factors in lymphoma, cytokines with increased secretion in co‐culture conditions upon copanlisib treatment that had not been well characterized for their role in the lymphoma field were selected for further analysis. Insulin‐like growth factor‐binding protein 3 (IGFBP‐3), Chitinase‐3‐like protein 1 (CHI3L‐1), Serpin E1, Pentraxin 3 (PTX‐3), Vascular Cell Adhesion Molecule 1 (VCAM1) and Dickkopf‐related protein 1 (DKK‐1) were supplemented as recombinant factors (Table S3) to stimulate VL51 cells before treatment with either Dimethyl sulfoxide (DMSO) or 10 nM copanlisib and cell viability was assessed using the 3‐[4,5‐dimethylthiazol‐2‐yl]‐2,5 diphenyl tetrazolium bromide (MTT) assay (copanlisib effect on viability shown in Figure 2E). Among the six cytokines, IGFBP‐3, Serpin E1 and PTX‐3 significantly reduced VL51 sensitivity to copanlisib (Figure 2F), indicating their possible role also in the VL51–BMSCs co‐culture. Previous studies have identified TMEM219 and TGFβR1 as the receptors for IGFBP‐3,^11^ urokinase plasminogen activator surface receptor (uPAR) as the receptor for Serpin E1^12^ and TLR4 as a receptor for PTX‐3.^13^ Immunofluorescence staining confirmed clear expression of all receptors in VL51 (Figure S6, details on used antibodies in Table S4), further supporting the potential involvement of IGFBP‐3, Serpin E1 or PTX‐3 in decreasing the sensitivity to copanlisib in the VL51 cell line.

To extend the potential clinical relevance of our findings, we tested whether IGFBP‐3, Serpin E1 and PTX‐3 could also reduce sensitivity to BTK inhibitors in additional B‐cell lymphoma cell lines. All the selected cell lines expressed the corresponding receptors TMEM219, TLR4, TGFβR1 and uPAR (Figure S6). Conversely, the analysis of the secretome by cytokine array revealed that none of the selected cell lines secreted IGFBP‐3, Serpin E1 or PTX‐3 at basal conditions (Figure S7A,B). All cell lines were then stimulated with the three cytokines. Cell viability was measured by MTT assay after 72 h of exposure to DMSO as a negative control or to different concentrations of ibrutinib among cells (ibrutinib effect on viability shown in Figure S8A, ibrutinib concentrations indicated in Table S5). Stimulation with IGFBP‐3, Serpin E1 or PTX‐3 (Table S3) did not confer a proliferative advantage to the cells (Figure S8B, light orange bars). However, a general trend of decreased sensitivity to ibrutinib was observed in all cell lines except for OCI‐Ly10, which showed increased sensitivity. A statistically significant reduction in ibrutinib sensitivity was observed in SSK41 upon IGFBP‐3 stimulation; in TMD8 upon IGFBP‐3, Serpin E1 and PTX‐3; and in MINO when stimulated with Serpin E1 (Figure S8B, dark orange bars). These findings underscore that the effects of IGFBP‐3, Serpin E1 and PTX‐3 on ibrutinib sensitivity are context‐ and cell line‐dependent.

In conclusion, we developed an in vitro 3D BM model for B‐cell lymphomas using a high‐throughput, automated and reproducible approach. The BM has long been recognized as a ‘privileged’ niche, providing a protective environment that shields lymphoma cells from chemotherapy effects.^5^, ^7^ Indeed, our 3D BM model showed decreased sensitivity of B‐cell lymphoma cells to BCR‐targeted agents. This study demonstrates that BMSCs reduce the sensitivity of MZL and MCL cells to PI3K and BTK inhibitors. Although the magnitude of these effects varied, being evident in some cases and modest or not statistically significant in others, the trend was consistent across cell lines and is in agreement with previous reports that implicate the role of BMSCs in drug resistance in haematological malignancies.^14^

We confirmed that some well‐described cytokines involved in lymphoma therapeutic resistance are secreted in our 3D co‐culture model, namely IL‐6 and CXCL12.^8^, ^15^ Particularly, targeting IL‐6 partially rescued VL51 sensitivity to copanlisib in the co‐culture. Moreover, our 3D co‐culture system was useful to identify novel cytokines (IGFBP‐3, PTX‐3 and Serpin E1) that might promote resistance to lymphoma treatments, though their exact roles and their clinical relevance require deeper investigation. The detection of the cytokines in the BMSC monoculture, but not in the lymphoma monocultures, suggests that BMSCs are the primary source. Nonetheless, we cannot exclude the contribution of additional BMSC‐derived factors in driving resistance, particularly in the context of BTK inhibitors and in the cell lines on which secretome analysis was not performed.

Despite its limitations, this study demonstrates important strengths. First, we developed a simplified in vitro 3D BM model that is automated, reproducible and scalable, allowing systematic interrogation of tumour–microenvironment interactions. Second, this study provides evidence that BMSCs might promote therapeutic resistance to PI3K and BTK inhibitors in MZL and MCL. Third, our model recapitulated established resistance mediators (IL‐6, CXCL12) while also identifying novel candidates (IGFBP‐3, Serpin E1, PTX‐3). Ultimately, the scalability of our model offers opportunities for subtype‐specific cytokine profiling and real‐time drug testing, with potential translational relevance for patients with relapsed and refractory lymphomas.

## AUTHOR CONTRIBUTIONS

A.Z., A.A., M.V.C., E.C. and A.A.M. performed the experiments. A.Z., A.A., L.C. analysedanalyzed the data and performed bioinformatic analysis. D.P. performed primary cell isolation. F.Si. provided mCherry‐positive cell lines. C.C. provided patient samples. F.Sp. and D.P. provided technical advice. A.Z., C.A., M.M. and F.B. designed the research and ideated the experiments. A.Z. wrote the manuscript. A.A., C.A., M.M. and F.B. revised the manuscript. All authors approved the final manuscript.

## CONFLICT OF INTEREST STATEMENT

L.C. institutional research funds from Orion; travel grant from HTG. A.A. travel grant from Astra Zeneca, consultant for PentixaPharm. F.B. institutional research funds from ADC Therapeutics, Bayer AG, BeiGene, Floratek Pharma, Helsinn, HTG Molecular Diagnostics, Ideogen AG, Idorsia Pharmaceuticals Ltd., Immagene, ImmunoGen, Menarini Recherche, Nordic Nanovector ASA, Oncternal Therapeutics, Spexis AG; consultancy fee from BIMINI Biotech, Helsinn, Menarini; advisory board fees to institution from Novartis; expert statements provided to HTG Molecular Diagnostics; travel grants from Amgen, Astra Zeneca, BeiGene, InnoCare, iOnctura. The other authors have nothing to disclose.

## Supporting information


Data S1.

## Data Availability

The data that support the findings of this study are available in the Supporting Information of this article.
