# Supplementary material for: A high‐throughput bone marrow 3D co‐culture system to study resistance to BCR signalling targeted agents in B‐NHL
Source: Br J Haematol. 2025 Dec 8;208(2):716–21. doi: 10.1111/bjh.70273 (PMC12916189; doi:10.1111/bjh.70273)
Supplement: Supplementary file 1 — Data S1. [file BJH-208-716-s001.zip › bjh70273-sup-0001-Supinfo.docx]

**A High-Throughput Bone Marrow 3D Co-Culture System to Study Resistance to BCR Signaling Targeted Agents in B-NHL**

Alex Zadro^1,2^, Alberto Arribas^2,3^, Maria Vittoria Colombo^1^, Eleonora Cannas^2^, Filippo Spriano^2^, Luciano Cascione^2,3^, Afua Adjeiwaa Mensah^2^, Federico Simonetta^4,5^, Dalila Petta^1^, Christian Candrian^6,7^, Chiara Arrigoni^1,7,#^, Francesco Bertoni^2,8 #,*^, Matteo Moretti^1,7,9,*^.

# CA, FB co-corresponding

* MM, FB equally contributing last authors

*^1^ Regenerative Medicine Division, Institute for Translational Research.,Università della Svizzera Italiana (USI) - Ente Ospedaliero Cantonale (EOC), Bellinzona, Switzerland*

*^2^ Institute of Oncology Research, Faculty of Biomedical Sciences, USI, Bellinzona, Switzerland*

*^3^ Swiss Institute of Bioinformatics, Lausanne, Switzerland;*

*^4^ Division of Hematology, Department of Oncology, Geneva University Hospitals, University of Geneva, Geneva, Switzerland*

*^5^ Translational Research Centre in Onco-Hematology, Faculty of Medicine, University of Geneva, Geneva, Switzerland*

*^6^ Service of Orthopedics and Traumatology, Department of Surgery, Ente Ospedaliero Cantonale (EOC), Lugano, Switzerland*

*^7^ Euler Institute, Faculty of Biomedical Sciences, USI, Lugano, Switzerland*

*^8^ Oncology Institute of Southern Switzerland, Ente Ospedaliero Cantonale (EOC), Bellinzona, Switzerland*

*^9^ IRCCS Istituto Ortopedico Galeazzi, Cell and Tissue Engineering Laboratory, 20161 Milano, Italy*

**Supplementary Appendix**

**Supplementary Materials and Methods**

*Primary cells*

The human-derived specimen sampling was conducted under the guidelines, regulations, and procedures of the Ente Ospedaliero Cantonale (Bellinzona, Switzerland) and approved by the local IRB (Approval n. 2020-00029 from Comitato Etico Cantonale). The biological samples used in the study are represented by waste surgical pieces, harvested from patients who signed an informed consent. All the individuals (four females, two males) were subjected to knee replacement surgery and presented OA Kellgren Lawrence grade 3 to 4, evaluated from radiological images. The age of the patients ranged between 58 and 73 years.

*Isolation of primary Bone Marrow Stromal Cells (BMSCs) and culture conditions*

Primary BMSCs were isolated from knee biopsies, as previously reported^1^. Following isolation, BMSCs were cryopreserved at -150°C, and all experiments were conducted within a maximum of three weeks after thawing. To create pools of BMSCs, equal numbers of cells from each donor were mixed. The pooled cells were then plated and cultured until they reached passage 3, at which point they were used for experiments.

BMSCs were cultured in MEM Alpha medium (12571063, Gibco) supplemented with 10% fetal bovine serum (FBS; F9665, Sigma-Aldrich), 1% HEPES (15630056, Gibco), 1% Penicillin-Streptomycin (P/S, 10,000 U/mL; 15140122, Gibco) and 5ng/mL basic fibroblasts growth factor (100-18B, Peprotech).

*Cell lines*

Established human cell lines derived from marginal zone lymphoma (VL51, K1718, SSK41, mCherry-VL51, mCherry-K1718 and mCherry-SSK41), diffuse large B-cell lymphoma (OCI-Ly-10 and TMD8), and mantle cell lymphoma (REC-1 and MINO) were cultured in RPMI 1640 medium supplemented with L-Glutamine (11875093, Gibco), 10% FBS and 1% P/S. mCherry-positive cell lines were established as previously described^2^. Cell line identity was authenticated by short tandem repeat DNA profiling, as previously described^3^. The cell lines were stored at -150°C, and all experiments were conducted within one to two months after thawing. Routine Mycoplasma testing was performed using the MycoStrip assay (rep-mysnc-100, InvivoGen) to confirm negativity.

*Seeding in 3D fibrin gel*

Cell-laden fibrin gels were prepared by suspending lymphoma cells and BMSCs in 4 U/mL thrombin (obtained from the TISSEEL Fibrin Sealant Kit, 025243179, Baxter) diluted in RPMI1640 supplement with L-glutamine, 10% FBS, and 1% P/S. This solution was then mixed with an equal volume of 40 mg/mL fibrinogen (F3879, Sigma) to achieve a final fibrin concentration of 20 mg/mL. After combining the fibrinogen and thrombin, the gels were allowed to polymerize at 37°C for 20 minutes. To maintain sterile conditions, automated high-throughput seeding was performed using the OT-2 liquid handler (Opentrons) equipped with a HEPA module. Cells were seeded into a final volume of 50 μL fibrin gels in Nunc MicroWell 96-Well plates (167008, Thermo Scientific).

*Copanlisib dose-response in 3D fibrin gel*

The mCherry-VL51 cells were seeded at 0.075 x 10^6^ cells/mL final concentration, the SSK41 and REC-1 cells were stained with Vybrant Did Cell-Labeling Solution (V22885, Invitrogen) according to manufacturer instructions and were seeded at 1 x 10^6^ cells/mL. All models were cultured either alone or in co-culture with primary BMSCs at 0.75 x 10^6^ cells/mL. Cells were maintained in RPMI-1640 medium supplemented with L-glutamine, 10% FBS, and 1% P/S, with a medium exchange performed on day 1. At day 3, specific treatment conditions, as indicated in the respective figures, were initiated. Copanlisib was purchased from Selleckchem (TX, USA) and prepared as stock solutions (10mM) in DMSO. On day 5, fresh treatment conditioned medium was added. At day 7, live cells were stained with calcein (C3099, Thermo Fisher). Live imaging of the cultures was performed on days 3, 5, and 7 using the Opera Phenix Plus High-Content Screening System (PerkinElmer) while keeping the plate at 37°C, 5% CO_2,_ and optimal humidity. The treatment effects were assessed through both morphological observations and quantitative image analysis. The growth curve for each condition was generated by measuring the total mCherry^+^ or Vybrant Did^+^ area for day 5 and measuring the total mCherry^+^ or Vybrant Did^+^ and calcein^+^ for day 7 from the maximum projection images, normalizing them first to the respective well at day 3, and then to the DMSO control for each condition (relative proliferation and relative viability, respectively).

*Treatment and cell proliferation evaluation in 3D fibrin gel*

The mCherry-VL51 cells were seeded at 0.075 x 10^6^ cells/mL final concentration, the mCherry-K1718 cells were seeded at 0.5 x 10^6^ cells/mL, the mCherry-SSK41 cells were seeded at 1 x 10^6^ cells/mL, the REC-1 and TMD8 cells were stained with Vybrant DiI Cell-Labeling Solution (V22885, Invitrogen) according to manufacturer instructions and were seeded at 1 and 0.15 x 10^6^ cells/mL, respectively. All models were cultured either alone or in co-culture with primary BMSCs, which had been pre-labeled using CFSE (CFSE Cell Labeling Kit, ab113853, Abcam), at 0.75 x 10^6^ cells/mL. Cells were maintained in RPMI-1640 medium supplemented with L-glutamine, 10% FBS, and 1% P/S, with a medium exchange performed on day 1. At day 3, specific treatment conditions, as indicated in the respective figures, were initiated. Ibrutinib was purchased from Selleckchem (TX, USA) and prepared as stock solutions (50mM) in DMSO. Sirukumab (A2980, Selleck Chemicals) and the human IgG1 isotype control (A2051, Selleck Chemicals) were used at 10µg/mL. On day 5, fresh treatment conditioned medium was added. Live imaging of the cultures was performed on days 3, 5, and 7 using the Opera Phenix Plus High-Content Screening System (PerkinElmer) while keeping the plate at 37°C, 5% CO_2,_ and optimal humidity. The treatment effects were assessed through both morphological observations and quantitative image analysis. The growth curve for each condition was generated by measuring the total mCherry^+^ or Vybrant Dil^+^ area from the maximum projection images, normalizing it first to the respective well at day 3, and then to the DMSO control for each condition (relative proliferation shown in percentage). Cluster size was determined by quantifying the mCherry^+^ or Vybrant Dil^+^ area, with the data representing the average of the fourth quartile from the cluster size distribution for each condition.

*Cytokine array*

Supernatants were collected from the different 3D experimental conditions at day 7 or after three days of culture (baseline expression) and were concentrated using the Amicon Ultra centrifugal filter units (Z648027, Merck). A 10X concentration was achieved by centrifugation at 16,000 rpm for 1h at 4°C. The concentrated media were then analyzed for 105 soluble human proteins using the Human XL Cytokine Array Kit (ARY022B, Biotechne) according to the manufacturer's protocol. The signals from the array membranes were captured using the Fusion Solo S imaging system (Vilber) and subsequently quantified using the Protein Array Analyzer plugin in ImageJ (NIH)^4^. The resulting data were normalized to the positive control signals on each membrane, and heatmaps were generated to represent cytokines’ relative secretion. Heatmap visualization was performed using the ComplexHeatmap package in R^5^, with values representing the averaged normalized signal for each cytokine across the experimental conditions.

*MTT assay*

Cells were incubated overnight (ON) in 1% FBS medium. Following incubation, cells were seeded into 96-well plates at the densities indicated for each cell line in *Supplementary Table 1*. Cells were cultured in either specific cytokine- or PBS-conditioned in 10% FBS media and stimulated at designated time points, with cytokine concentrations and stimulation durations detailed in *Supplementary Table 1*. All recombinant cytokines used in the experiments were purchased from ProSpec-Tany Technogene (Israel). After stimulation, plates were centrifuged at 1,200 rpm for 5 minutes to remove the conditioned media, and the cell pellets were resuspended in either DMSO or drug-conditioned, phenol red-free media. The specific drug and its concentration for each cell line are indicated in the figures and *Supplementary Table 2*. Cells were incubated under these conditions for 72 hours followed by an MTT assay as previously described^6^.

*Cytokines receptors immunofluorescence*

The IbiTreat µ-Slide 8 Well (80826, Ibidi) and IbiTreat µ-Slide 18 Well (81816, Ibidi) were pre-coated by applying poly-L-ornithine solution (A-004-C, Sigma) to each well and incubating at room temperature (RT) for 1 hour. After coating, the solution was removed, and the plates were allowed to air dry. Cells were then seeded in 1% FBS medium at 1 x 10^5^ cells per well (100 µL of a 1 x 10^6^ cells/mL suspension) and allowed to attach ON in the incubator. The media was removed the following day, and the wells were gently washed once with DPBS with calcium and magnesium (14040117, Gibco). Cells were fixed with 4% paraformaldehyde (PFA, 1004965000, Merck) for 15 minutes at RT, followed by three washes with DPBS, each lasting 5 minutes. To block non-specific binding, 4% bovine serum albumin (BSA) in DPBS was added and incubated for 1 hour at RT while shaking. After blocking, the BSA solution was removed, and primary antibodies (more details in *Supplementary Table 3*), diluted in 4% BSA, were added for overnight incubation at 4°C. The next day, wells were washed three times with DPBS for 5 minutes. Secondary antibodies (more details in *Supplementary Table 3*), diluted in 4% BSA with 1:1000 NucBlue Fixed Cell ReadyProbes Reagent (R37606, Invitrogen), were added and incubated for 1 hour at RT. Following secondary antibody incubation, wells were washed three times with DPBS for 5 minutes. Samples were then stored in DPBS containing 0.1% sodium azide (71289-50G, Sigma Aldrich) for long-term preservation at 4°C.

*Graphical representation and statistical analysis*

Graphical representation and statistical analyses were performed using GraphPad Prism version 8.01 (https://www.graphpad.com), unless stated otherwise. The specific statistical tests used are detailed in the respective figure legends.

*Supplementary Table 1*

Ibrutinib concentration used in in 3D fibrin gel for the different cell lines.

| **Cell line** | **Ibrutinib concentration (nM)** |
| --- | --- |
| VL51 | 5000 |
| Karpas1718 | 50 |
| SSK41 | 100 |
| TMD8 | 25 |
| REC-1 | 100 |

*Supplementary Table 2*

Pannel of cytokines detected by cytokine array. Further details indicated in the *Material and Methods* section.

| **Coordinate** | **Cytokine** | **Alias** |
| --- | --- | --- |
| A1-A2 | positive cnt |  |
| A3-A4 | Adinopectin |  |
| A5-A6 | Apolopoprotein 1 |  |
| A7-A8 | Angiogenin |  |
| A9-A10 | Angiopoietin-1 |  |
| A11-A12 | Angiopoietin-2 |  |
| A13-A14 | BAFF |  |
| A15-A16 | BDNF |  |
| A17-A18 | Complement component C5 |  |
| A19-A20 | CD14 |  |
| A21-A22 | CD30 |  |
| A23-A24 | positive cnt |  |
| B1-B2 |  |  |
| B3-B4 | CD40 ligand |  |
| B5-B6 | Chitinase 3-like 1 |  |
| B7-B8 | Complement Factor D |  |
| B9-B10 | C-Reactive Protein |  |
| B11-B12 | Cripto-1 |  |
| B13-B14 | Cystatin C |  |
| B15-B16 | Dkk-1 |  |
| B17-B18 | CD26 | DPP4 |
| B19-B20 | EGF |  |
| B21-B22 | CD147 | Emmprin |
| B23-B24 |  |  |
| C1-C2 |  |  |
| C3-C4 | CXCL5 |  |
| C5-C6 | ENG | CD105, endoglin |
| C7-C8 | TNSF6 | FAS ligand, CD178, CD95L |
| C9-C10 | FGF-2 |  |
| C11-C12 | FGF-7 |  |
| C13-C14 | FGF-19 |  |
| C15-C16 | FLT3LG | FLT-3 ligand |
| C17-C18 | G-CSF | CSF3 |
| C19-C20 | MIC-1 | GDF-15 |
| C21-C22 | CSF2 | GM-CSF |
| C23-C24 |  |  |
| D1-D2 | CXCL1 | GROa, MSGA-a |
| D3-D4 | GH | Growth Hormone |
| D5-D6 | HGF | SF, Scatter Factor |
| D7-D8 | ICAM-1 | CD54 |
| D9-D10 | IFNG | IFN-gamma |
| D11-D12 | IGFBP-2 |  |
| D13-D14 | IGFBP-3 |  |
| D15-D16 | IL1a | IL-1a |
| D17-D18 | IL1b | IL-1b |
| D19-D20 | IL1ra | IL1-ra |
| D21-D22 | IL2 | IL-2 |
| D23-D24 | IL3 | IL-3 |
| E1-E2 | IL4 | IL-4 |
| E3-E4 | IL5 | IL-5 |
| E5-E6 | IL6 | IL-6 |
| E7-E8 | IL8 | IL-8 |
| E9-E10 | IL10 | IL-10 |
| E11-E12 | IL11 | IL-11 |
| E13-E14 | IL12 p70 | IL-12 p70 |
| E15-E16 | IL13 | IL-13 |
| E17-E18 | IL15 | IL-15 |
| E19-E20 | IL16 | IL-16 |
| E21-E22 | IL17a | IL-17A, CTLA8 |
| E23-E24 | IL18 Bpa | IL-18 Bpa |
| F1-F2 | IL19 | IL-19 |
| F3-F4 | IL22 | IL-22. IL-TIF |
| F5-F6 | IL23 | IL-23, IL-23A, SGRF |
| F7-F8 | IL24 | IL-24, C49A, FISP, MDA-7, MOB-5, ST16 |
| F9-F10 | IL27 | IL-27 |
| F11-F12 | IL31 | IL-31 |
| F13-F14 | IL32 | IL-32 |
| F15-F16 | IL33 | IL-33 |
| F17-F18 | IL34 | IL-34 |
| F19-F20 | CXCL10 | IP-10 |
| F21-F22 | CXCL11 | I-TAC, SCYB9B |
| F23-F24 | KLK3 | Kallikrein, PSA |
| G1-G2 | Leptin | OB |
| G3-G4 | LIF |  |
| G5-G6 | Lipocaliln-2 | NGAL, LCN2, Siderocalin |
| G7-G8 | CCL2 | MCP-1, MCAF |
| G9-G10 | CCL7 | MCP-3, MARC |
| G11-G12 | CSF1 | M-CSF |
| G13-G14 | MIF |  |
| G15-G16 | CXCL9 | MIG |
| G17-G18 | CCL3 | CCL3/CCL4, MIP-1a/MIP-1b |
| G19-G20 | CCL20 | MIP-3a, Exodus-1, LARC |
| G21-G22 | CCL19 | MIP-3b, ELC |
| G23-G24 | MMP-9 | CLG4B, Gelatinase B |
| H1-H2 | MPO | Myeloperoxidase, Lactooperoxidase |
| H3-H4 | OPN | Osteopontin |
| H5-H6 | PDGF-AA |  |
| H7-H8 | PDGF-AB/BB |  |
| H9-H10 | PTX3 | Pentraxin 3, TSG-14 |
| H11-H12 | CXCL4 | PF4 |
| H13-H14 | RAGE |  |
| H15-H16 | CCL5 | RANTES |
| H17-H18 | RBP-4 |  |
| H19-H20 | RLN2 | Relaxin-2, RLXH2 |
| H21-H22 | Resistin | RETN, ADSF, FIZZ3 |
| H23-H24 | CXCL12 | SDF-1a, PBSF |
| I1-I2 | Serpin E1 | PAI-I, PAI-1, Nexin |
| I3-I4 | SHBG | ABP |
| I5-I6 | IL1R-L1 | IL-1 R4, IL1RL1, ST2, ST2L |
| I7-I8 | CCL17 | TARC |
| I9-I10 | TFF3 | ITF, TFI |
| I11-I12 | CD71 | TfR, TFR1, TFRC, TRFR |
| I13-I14 | TGF-a | TGFA |
| I15-I16 | Thrombospondin-1 | THBS1, TSP-1 |
| I17-I18 | TNF-a | TNFSF1A |
| I19-I20 | uPAR | PLAUR |
| I21-I22 | VEGF | BEGFA |
| I23-I24 |  |  |
| J1-J2 | positive cnt |  |
| J3-J4 |  |  |
| J5-J6 | Vitamine D BP | VDB, DBP, VDBP |
| J7-J8 | CD31 | PECAM-1 |
| J9-J10 | TIM-3 | HAVCR2 |
| J11-J12 | VCAM-1 | CD106 |
| J13-J14 |  |  |
| J15-J16 |  |  |
| J17-J18 |  |  |
| J19-J20 |  |  |
| J21-J22 |  |  |
| J23-J24 | negative cnt |  |

*Supplementary Table 3*

Cell seeding concentrations and cytokine stimulation conditions used for MTT assays as indicated in the *Material and Methods* section.

| **Cell Line** | **Seeding Concentration**  **(cells/well)** | **CHI3L1** | **VCAM** | **DKK1** | **IGFBP-3** | **Serpin E1** | **PTX-3** |
| --- | --- | --- | --- | --- | --- | --- | --- |
|  |  | **Concentration (ng/mL), Time** | | | | | |
| VL51 | 1 x 10^4^ | 1, 4h | 1, 4h | 1, 4h | 1, 4h | 1, 4h | 1, 4h |
| Karpas1718 | 2 x 10^4^ |  | | | 10, 24h | 1, 24h | 100, 4h |
| SSK41 | 2 x 10^4^ |  |  |  | 10, 1h | 100, 24h | 100, 4h |
| OCI-Ly10 | 2 x 10^4^ |  |  |  | 1, 4h | 1, 1h | 1, 1h |
| TMD8 | 1 x 10^4^ |  |  |  | 1, 24h | 1, 4h | 1, 1h |
| REC-1 | 2 x 10^4^ |  |  |  | 100, 4h | 1, 1h | 100, 24h |
| MINO | 1 x 10^4^ |  |  |  | 100, 4h | 100, 4h | 100, 1h |

*Supplementary Table 4*

Antibody list used for IF as indicated in the *Material and Methods* section.

| **Antibody** | **Catalog Number** | **Type** | **Host** | **Dilution Used** |
| --- | --- | --- | --- | --- |
| TLR4 Polyclonal Ab | bs-1201R | Primary | Rabbit | 1:200 |
| TGF beta R1 Polyclonal Ab | bs-0638R | Primary | Rabbit | 1:200 |
| Anti-TMEM219 | HPA059185 | Primary | Rabbit | 1:200 |
| uPAR recombinant mouse monoclonal Ab (2E2) | MA5-50263 | Primary | Mouse | 1:200 |
| Anti-Mouse Alexa Fluor™ 488 | A-28175 | Secondary | Goat | 1:1000 |
| Anti-Rabbit Alexa Fluor™ 488 | A-11008 | Secondary | Goat | 1:1000 |

*Supplementary Table 5*

Drug concentrations used for MTT assays as indicated in the *Material and Methods* section.

| **Cell Line** | **Copanlisib** | **Ibrutinib** |
| --- | --- | --- |
|  | **Concentration (nM)** | |
| VL51 | 10 |  |
| Karpas1718 |  | 1 |
| SSK41 |  | 1 |
| OCI-Ly10 |  | 0.5 |
| TMD8 |  | 0.5 |
| REC-1 |  | 2 |
| MINO |  | 10 |

**References**

1 D. Petta, D. D’Arrigo, S. Salehi, et al. A personalized osteoarthritic joint-on-a-chip as a screening platform for biological treatments. *Materials Today. Bio*. 2024; 26: 101072.

2 S. Wang, A. J. Arribas, C. Tarantelli, et al. PI3K and btk inhibition induces the upregulation of cd19 and increases sensitivity to car t cells in a model of marginal zone lymphoma (mzl). *Blood*. 2022; 140(Supplement 1): 4554–4555.

3 F. Spriano, E. Y. L. Chung, E. Gaudio, et al. The ets inhibitors yk-4-279 and tk-216 are novel antilymphoma agents. *Clinical Cancer Research*. 2019; 25(16): 5167–5176.

4 C. A. Schneider, W. S. Rasband, & K. W. Eliceiri. NIH image to imagej: 25 years of image analysis. *Nature Methods*. 2012; 9(7): 671–675.

5 Z. Gu, R. Eils, & M. Schlesner. Complex heatmaps reveal patterns and correlations in multidimensional genomic data. *Bioinformatics (Oxford, England)*. 2016; 32(18): 2847–2849.

6 S. W. Hicks, C. Tarantelli, A. Wilhem, et al. The novel cd19-targeting antibody-drug conjugate hub4-dgn462 shows improved anti-tumor activity compared to sar3419 in cd19-positive lymphoma and leukemia models. *Haematologica*. 2019; 104(8): 1633–1639.

**Supplementary Figures**


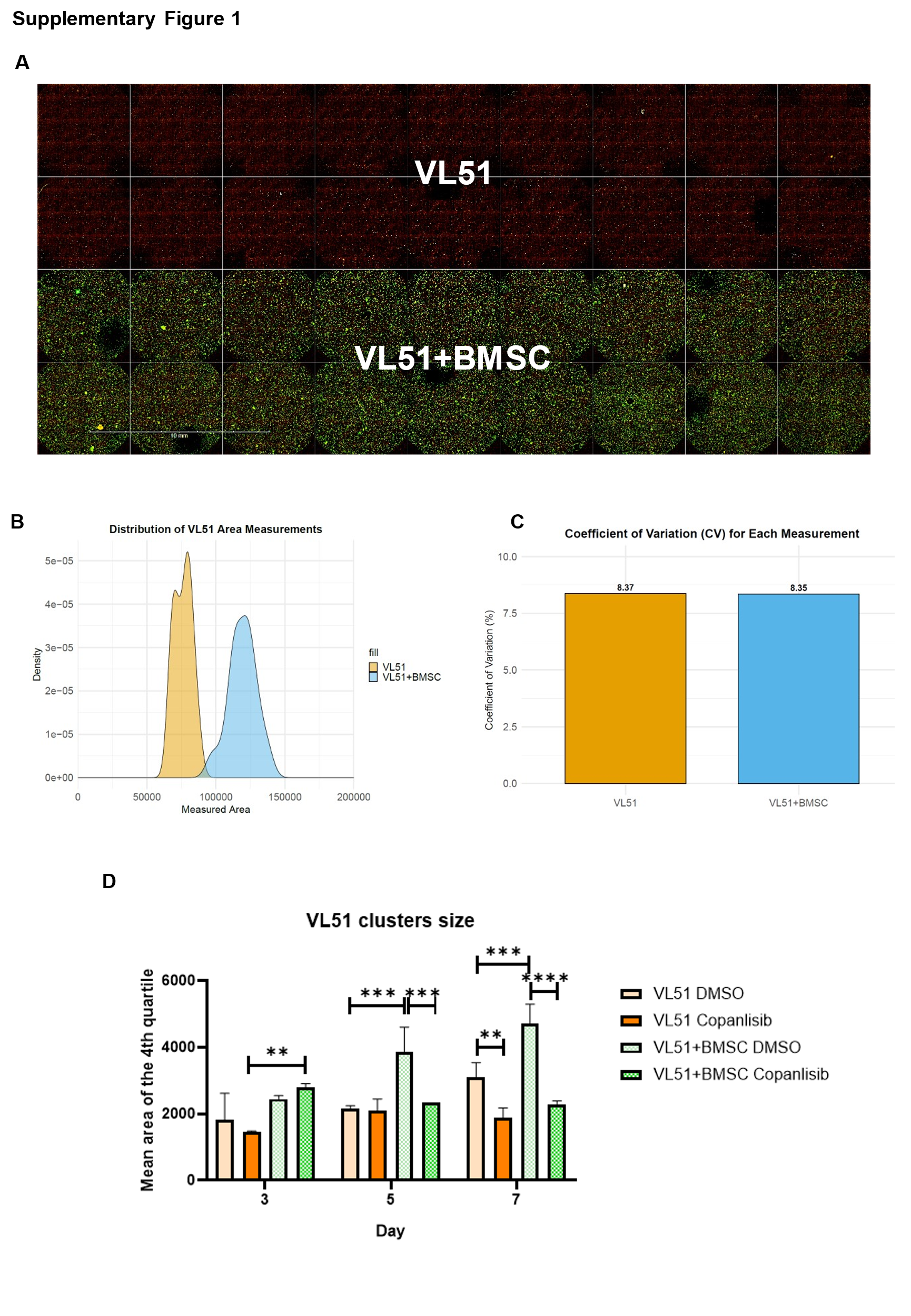


**Supplementary Figure 1**

1. Representative immunofluorescence maximum projection images of VL51 (in orange) cultured in the presence or absence of BMSCs (in green) on day 0 post-seeding. Images taken with 10x Air objective NA 0.3, WD 5.2mm of the Opera Phenix Plus High-Content Screening System while keeping the plate at 37°C, 5% CO_2,_ and optimal humidity. Scale bar: 10mm.
2. Plot showing the distribution of the VL51 measured areas (mCherry positive) on maximum projection images (Supplementary Figure 1A) between wells and among mono- (orange) and co-culture (light blue) conditions. Plot generated with R.
3. Bar plot showing the coefficient of variation of the VL51 measured areas (mCherry potisitive) on maximum projection images (Supplementary Figure 1A) between wells and among mono- (orange) and co-culture (light blue) conditions. Plot generated with R.
4. Quantification of VL51 cluster size on maximum projection images. The barplot shows the averaged values with standard deviation in the 4th quartile of the distribution of the measured areas of three independent biological replicates. Statistical significance tested with Two-way ANOVA + Dunnett's multiple comparisons test (* = p < 0.05 , ** = p < 0.01 , *** = p < 0.001).


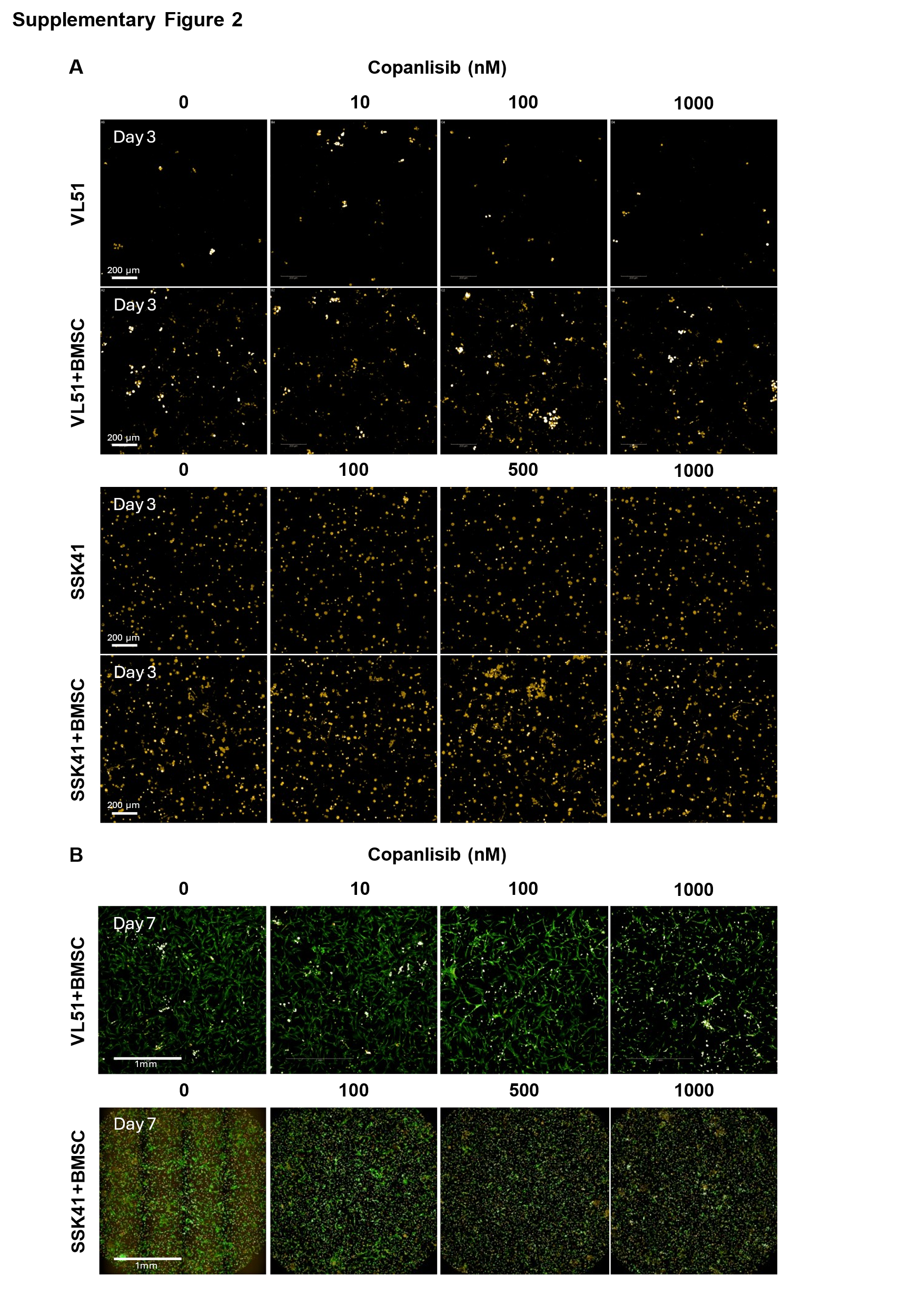


**Supplementary Figure 2**

1. Representative immunofluorescence maximum projection images of the indicated B cell lymphoma cell lines (in orange) cultured in the presence or absence of BMSCs on day 3. Images taken with 10x Air objective NA 0.3, WD 5.2mm of the Opera Phenix Plus High-Content Screening System while keeping the plate at 37°C, 5% CO_2,_ and optimal humidity. Scale bar: 200μm.
2. Representative immunofluorescence maximum projection images of the indicated B cell lymphoma cell lines (in orange) cultured in the presence of BMSCs (in green) on day 7, under the indicated culture conditions. Images taken with 10x Air objective NA 0.3, WD 5.2mm of the Opera Phenix Plus High-Content Screening System while keeping the plate at 37°C, 5% CO_2,_ and optimal humidity. Scale bar: 1mm.


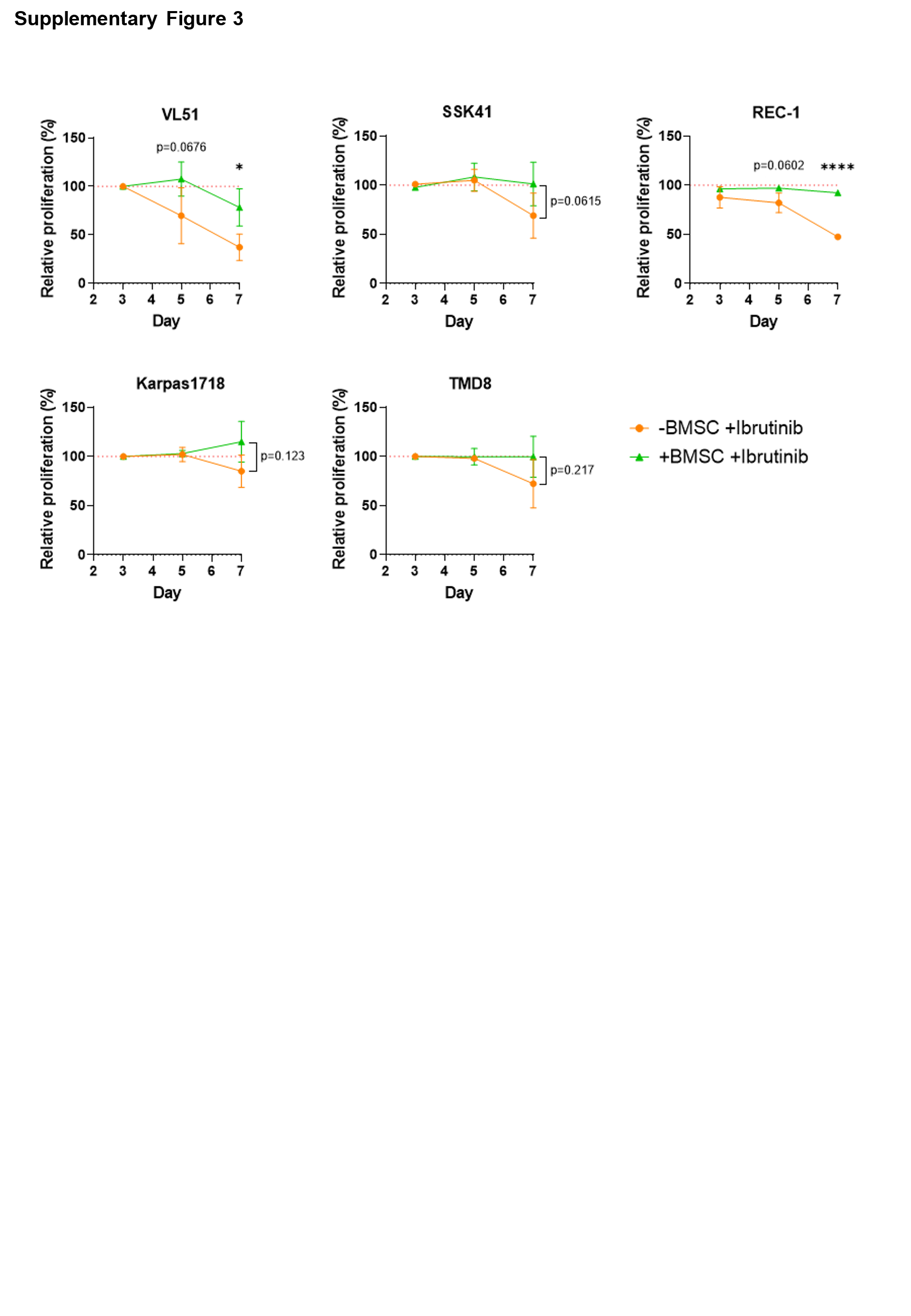


**Supplementary Figure 3**

1. Relative proliferation compared to the respective control of the indicated cell lines in mono- or co-culture upon DMSO or ibrutinib at day 3, 5 and 7. Values are plotted as the mean with standard deviation of at least three independent biological replicates. Statistical significance tested with Multiple t test (* = p < 0.05 , ** = p < 0.01 , *** = p < 0.001, **** = p < 0.0001 ).


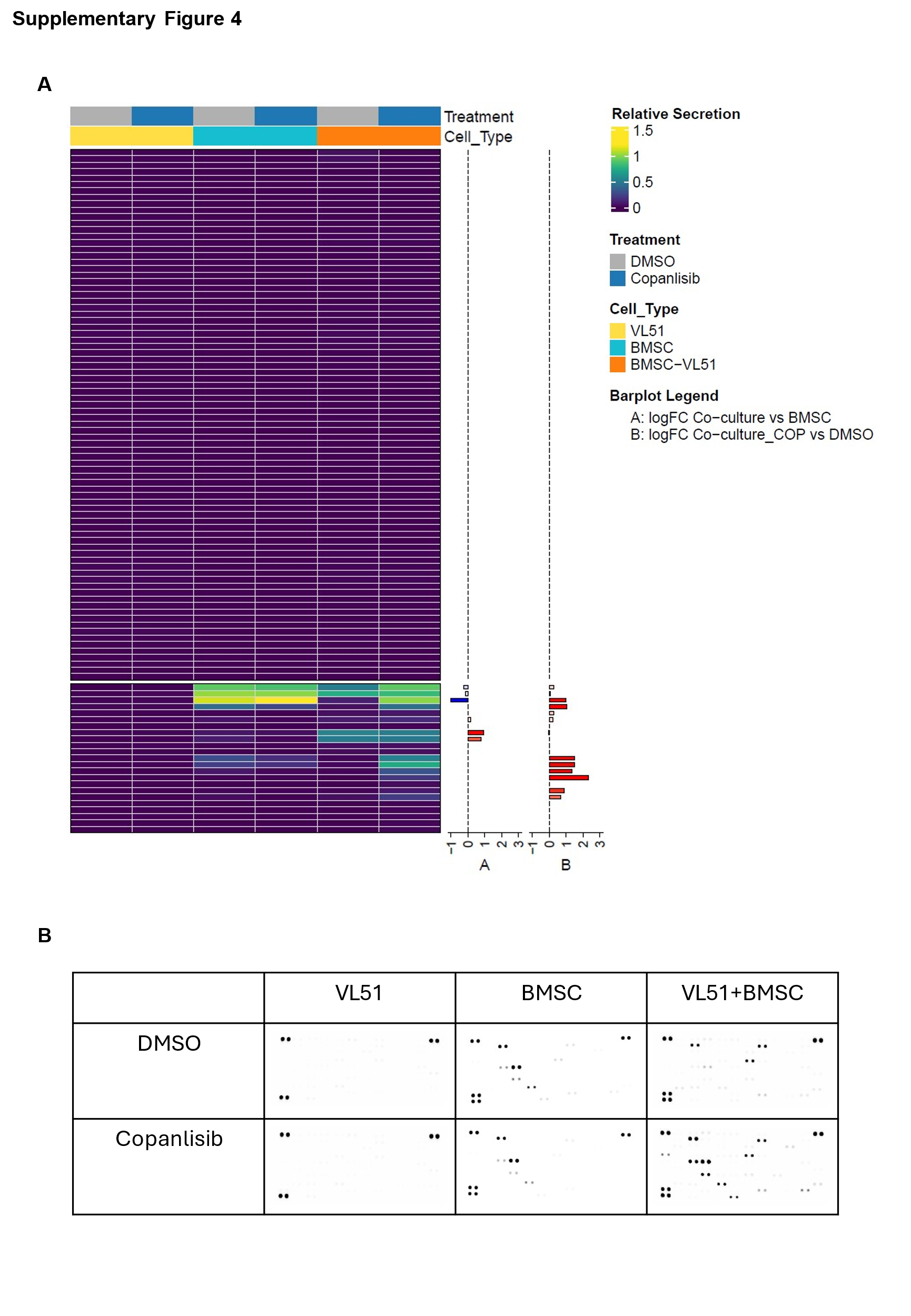


**Supplementary Figure 4**

1. Plot showing the whole panel of cytokines analyzed with the cytokine array. The heatmap was generated with the relative secretion values (row values normalized to internal positive and negative controls) of each cytokine in the different culture conditions. The bar plots show the log fold change values comparing co-culture to BMSC mono-culture upon DMSO (A) or the copanlisib to the DMSO conditions in the co-culture (B).
2. Representative images of the cytokine array membranes of all conditions.


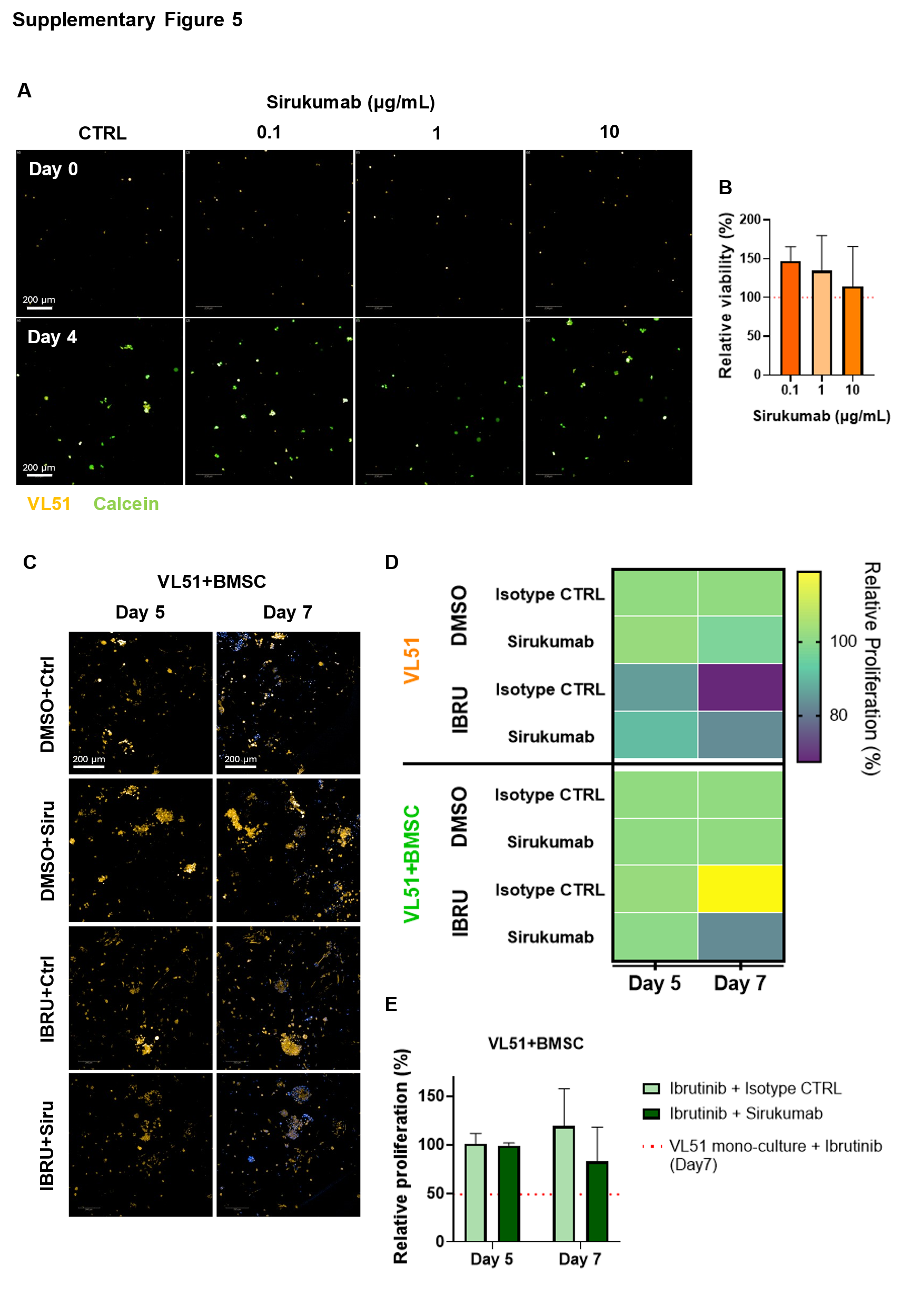


**Supplementary Figure 5**

1. Representative immunofluorescence maximum projection images of the VL51 cell line (in orange) treated with different single-agent sirukumab concentrations. Images taken with 10x Air objective NA 0.3, WD 5.2mm of the Opera Phenix Plus High-Content Screening System while keeping the plate at 37°C, 5% CO_2,_ and optimal humidity. Scale bar: 200μm.
2. Bar plot showing the quantification of the mCherry and calcein positive area on maximum projection images, representative of live VL51 occupied volume. The measurements shown were normalized to the day 0 time point, and the co-culture-treated conditions were normalized to the respective isotype control. Values are plotted as mean with standard deviation of three technical replicates and represent the relative viability of cells in the indicated condition compared to the control.
3. Representative immunofluorescence maximum projection images of VL51 (in orange) cultured in the presence of BMSCs upon DMSO+isotype, DMSO+sirukumab, ibrutinib+isotype CTRL or ibrutinib+sirukumab treatment on day 5 and 7 (ibrutinib concentration=5µM, sirukumab concentration=10μg/mL). Nuclei stained with Hoechst (in blue). Images taken with 10x Air objective NA 0.3, WD 5.2mm of the Opera Phenix Plus High-Content Screening System while keeping the plate at 37°C, 5% CO2, and optimal humidity. Scale bar: 200μm.
4. Plot showing the response of VL51 in mono- and co-culture to ibrutinib with or without sirukumab. The heatmap was generated with the relative proliferation values of each culture condition at day 5 and 7.
5. Bar plot showing the quantification of the mCherry or Vybrant Did positive area on maximum projection images, representative of VL51 occupied volume. The measurements shown were normalized to the day 3 time point and the co-culture treated conditions were normalized to respective DMSO control. Values are plotted as mean with standard deviation of at least three independent biological replicates and represents the relative proliferation of cells in the indicated condition compared to the control. Statistical significance was tested with a Multiple t-test. The dotted red line represents the proliferation of VL51 in mono-culture upon ibrutinib+isotype CTRL (ibrutinib concentration=5µM).

**
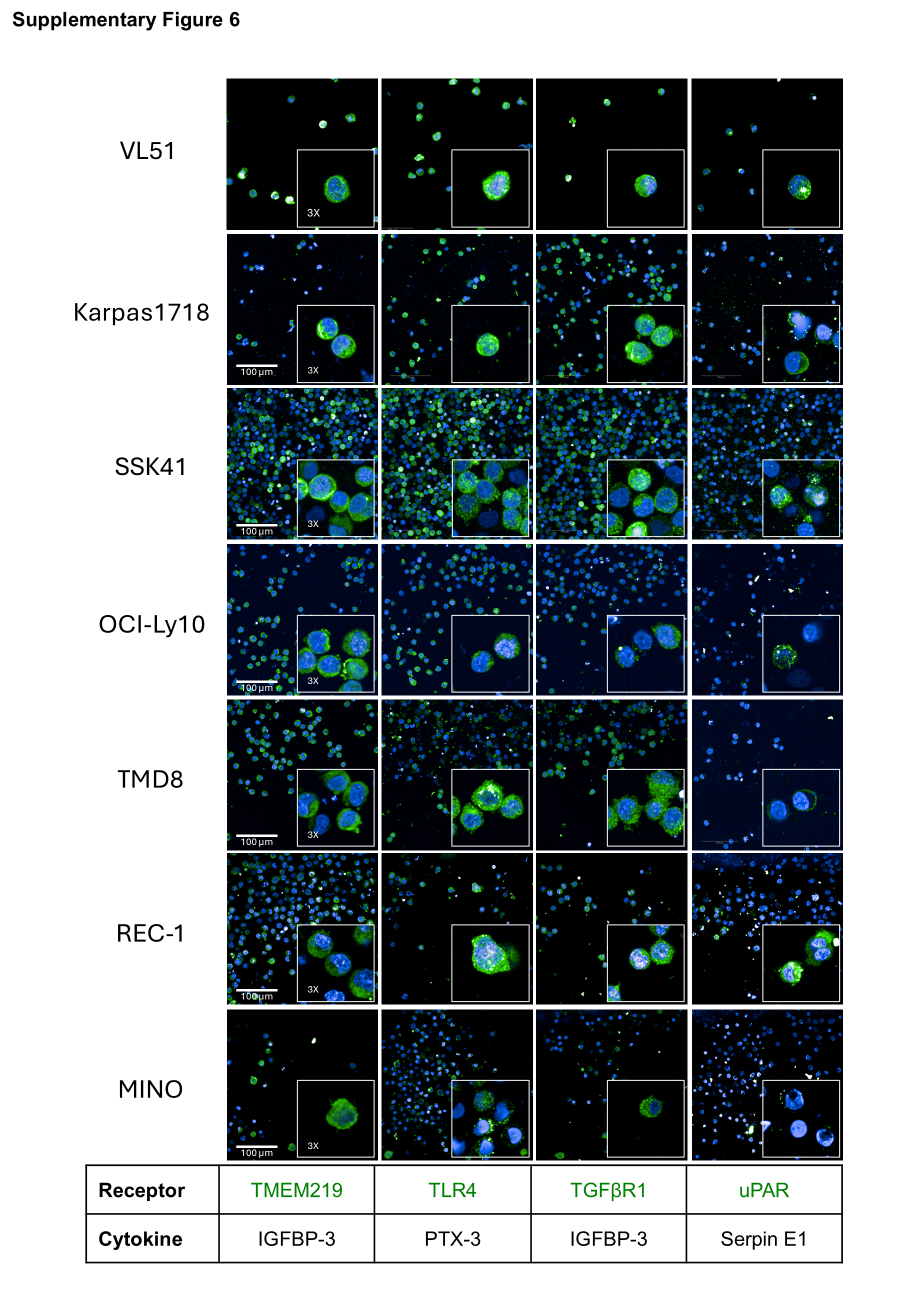
**

**Supplementary Figure 6**

1. Representative immunofluorescence images of the indicated cell lines. In green, it is the expression of the specified receptor, and, in blue, the nuclei. Images taken with 40x Water objective NA 1.1, WD 0.62mm of the Opera Phenix Plus High-Content Screening System at room temperature.


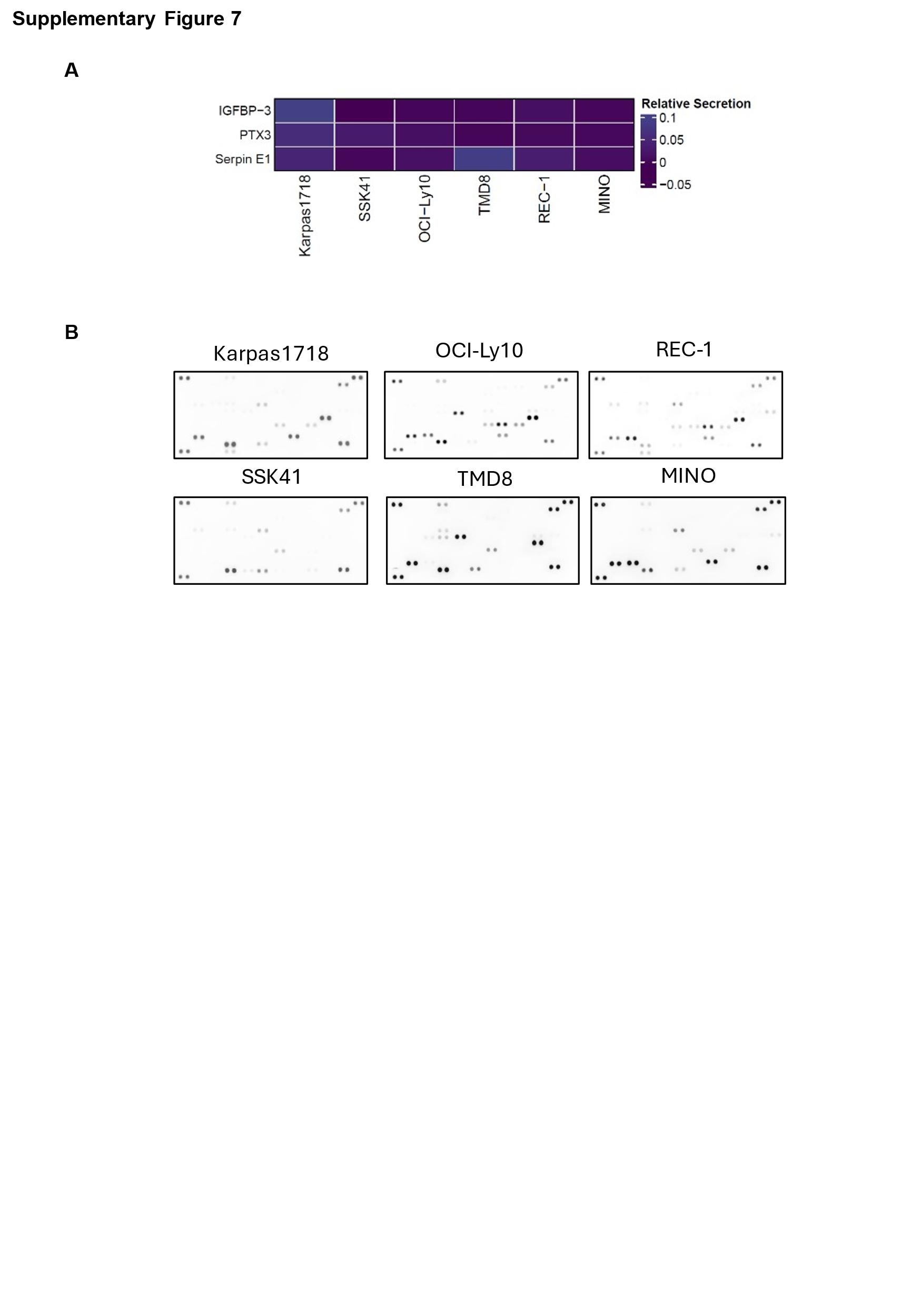
 **Supplementary Figure 7**

1. Plot showing IGFBP-3, PTX-3, and Serpin E1 secretion analyzed with the cytokine array. The heatmap was generated with the relative secretion values (row values normalized to internal positive and negative controls) of each cytokine in the indicated cell lines.
2. Representative images of the cytokine array membranes of all conditions.


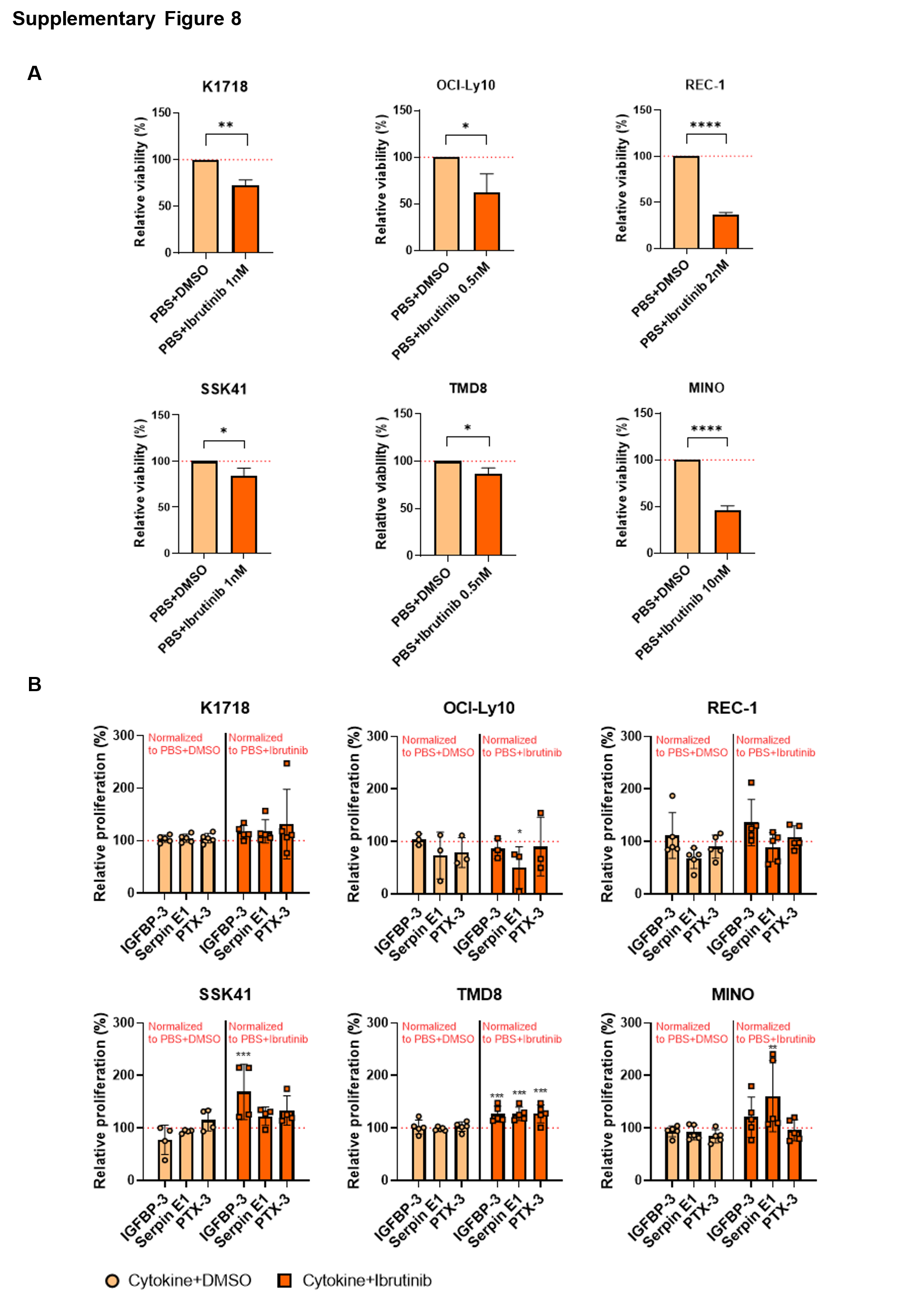


**Supplementary Figure 8**

1. Bar plot showing the effect of the ibrutinib concentrations on the indicated cells viability tested with MTT assay. The plotted values represent the mean with a standard deviation of at least three independent biological replicates. Statistical significance tested with Multiple t test (* = p < 0.05 , ** = p < 0.01 , *** = p < 0.001).
2. Bar plot representing the proliferation of indicated cell lines tested with MTT assay under the different culture conditions. The cytokine-stimulated conditions upon DMSO normalized to the respective PBS+DMSO control, representative of the cytokine-driven proliferative advantage, are plotted in light orange. The cytokine-stimulated conditions upon ibrutinib, normalized to the respective PBS+ibrutinib control, representative of the cytokines’ influence on ibrutinib sensitivity, are plotted in dark orange. The plotted values represent the mean with standard deviation of at least three independent biological replicates. Statistical significance tested with Two-way ANOVA + Dunnett's multiple comparisons test (* = p < 0.05 , ** = p < 0.01 , *** = p < 0.001).
